# Progress in Dermatology

**Published:** 1901-10-26

**Authors:** 


### PROGRESS IN DERMATOLOGY.

(*Continued from page* 53.)

**Eczema.**—Sarcoma of the skin comprises a large and ill-defined group of affections. J. C. Johnston [11] divides it into three classes. The first includes spindle and round cell tumours originating from fibroblasts. Giant cell forms are not primary in the skin. The second group includes lymphoid cell growths, such as are seen in Hodgkin's disease and lymphatic leukæmia. These form in the skin true tumours, but there is no inter-cellular substance present as a product of the cells. There are no vessels running between them, and the normal tissues do not melt away before them. Thirdly, we have the sarcoid tumours which present a reticulum, but never produce metastases, and most forms of which are curable by arsenic. Of this class are the cases of multiple pigmented sarcoma. We must distinguish *multiple cutaneous fibromata*, which are in many cases true neuro-fibromata, as Von Recklinghausen argued. Preble and Hektoen [12] discuss an instance in a woman who also suffered from arthritis deformans. A large number of tumours existed from the size of a pin's head upwards, and before death, which occurred without any definite cause, gangrene of the toes was noticed. It was found that many of the spinal and sympathetic nerves were marked by beaded enlargements, while in the cutaneous tumours fibrous tissue existed throughout the peripheral nerve bundles and the perineurium was thickened. The authors, however, consider that multiple fibromata do not in all cases arise from the fibrous tissue surrounding the nerves. Galloway remarks that multiple myomata may be easily mistaken for these fibrous tumours, and quotes a case where they were scattered over the right leg and the sternum, and accompanied with paroxysmal spontaneous pain.

**Tropical Diseases.**—*Fibroid tumours* are very apt to develop in the skin of negro races, and the tendency is often utilised in tattooing. Le Danteg and Boyé [13] have made a study of these tumours in the lobe of the ear which frequently follow piercing. They prove to be true fibromas, containing nothing but connective tissue, and not exuberant keloids, though they develop after a wound. *Prickly heat.*—R. R. H. Moore [14] lays down that this may be avoided by not using soap in one's bath, and by anointing the body with fresh sweet cocoanut oil, or with almond oil and lanoline as recommended by F. Pearse. The oil should be rubbed in daily before the evening exercise. If, however, a sponge be used for applying it, or the clothes be soaked with it, a smell will soon be developed as it turns rancid. When proper friction, however, is applied, there is no odour, and the skin remains healthy. When the skin is constantly wet from perspiration, its natural protection is the product of the sebaceous glands, and if this is constantly removed by soap, the result is a dermatitis. *Water itch*, affecting the feet of coolies working in tea-gardens during the wet months, causes a large amount of incapacity for work. A vesicular dermatitis appears, becomes pustular, and often leaves troublesome ulcers. A. B. Dalgetty[15] traces the life history of the acarus which produces it, and which seems to be a rhizoglyphus mite normally living on decaying matter, but becoming parasitic under favourable circumstances. Owing to the habits of the coolies, the ground becomes soaked with fæcal matter after a year or two, and as the majority are bare-footed they quickly take any infection from the wet soil. *Epidermolysis bullosa*, as a hereditary disease, is rare even in white races. H. L. Smith[16] has recorded the first known instance in a coloured person. The patient, his father and grandmother, suffered from frequent outbreaks of water-blisters on the hands, feet, buttocks, and scalp, especially in warm weather. After a week's duration they would break and dry up, leaving merely a brownish discoloration. The blood showed an excess of eosinophile corpuscles.

**Dermatitis.**—Newborn[17] discusses the acute dermatitis caused by a much-used hair-dye, paraphenylene diamine. The eruption may occur even in distant parts of the body, but causes especially a rash in the upper third of the face, with œdema of the eyelids and vesicles on the ears. There is often much itching and prickling in the eyes. The dye is contained in two liquids, a dark and a clear one which are applied in succession. Sedative applications are indicated, and in severe cases the hair should be cut off. *Orthoform*, though very insoluble, may, when applied in large quantities over a raw surface, give rise to toxic symptoms and eruptions. Dubreuilh[18] records the occurrence of erythematous rashes with or without vesicles or pustules, and more rarely of gangrene after its use.

**Treatment.**—Sachs[19] writes in favour of peruol in the cure of *scabies*, because its destructive action on the parasite is certain; it has no bad effect on the skin or on the internal organs; it is odourless, and does not discolour the skin or clothes. The entire body is rubbed with it three times in 36 hours After the last rubbing the bed and underclothing are changed, and after three days a soap bath is given. If no irritation of the skin existed beforehand it is well to give a bath before commencing treatment. Children bear the application very well. *Senile pruritus* may be alleviated by brushing with a soft brush, according to Jaenecke,[20] at first three times a day, and afterwards at rarer intervals. No water should be employed, but a little lanolin applied between the brushings, which should last from 10 minutes to half an hour. This removes the degenerated epithelial cells and increases the nutrition of the part, so that after a short time the patient finds that much less itching comes on and he may at length be quite freed from suffering. For *molluscum contagiosum* Balzer and Alquice[21] recommend breaking open the little tumours and inserting a splinter dipped in a tincture of iodine. When they are large this should be pushed about in various directions. The iodine dries up the mass, which soon scales off or may be removed by a curette.

[11] Brit. Jour. Dermatol., July. [12] Practit., May, p. 572. [13] Jour. Trop. Med., May 1. [14] Ibid., p. 142. [15] Jour. Trop Med., March 1. [16] Lancet, April 27. [17] Lancet, June 29. [18] Brit. Jour. Dermatol., July. [19] Therapeut., March 15. [20] Treatment, March, p. 34. [21] Edin. Med. Jour., July.

